# Effect of early use of ivabradine on left ventricular remodeling after primary percutaneous coronary intervention in patients with acute ST‐segment elevation myocardial infarction: A pilot test

**DOI:** 10.1111/anec.12816

**Published:** 2020-12-24

**Authors:** Yan Xu, Wenying Zhang, Xinbo Zhong, Shaodi Yan, Haijun Chen, Ruirui Guo, Xinlin Luo, Qiang Liu

**Affiliations:** ^1^ Department of Cardiology Fuwai Hospital Chinese Academy of Medical Sciences Shenzhen China; ^2^ Department of Pharmacy Fuwai Hospital Chinese Academy of Medical Sciences Shenzhen China; ^3^ Department of Echocardiography Fuwai Hospital Chinese Academy of Medical Sciences Shenzhen China

**Keywords:** emergency, Ivabradine, left ventricular remodeling, myocardial infarction, percutaneous coronary intervention (PCI)

## Abstract

**Objective:**

To investigate the effect of early use of ivabradine on left ventricular remodeling after primary percutaneous coronary intervention (PCI) in patients with acute ST‐segment elevation myocardial infarction (STEMI).

**Methods:**

A total of 66 STEMI patients with sinus rhythm and the resting heart rate ≥80 bpm after successful emergency PCI were included. The patients in the test group were treated with ivabradine combined with metoprolol at 12 hr after PCI, while the control group was given only metoprolol orally. Their resting heart rate was controlled to <70 bpm at discharge and followed for 180 days. Heart rate and blood pressure were measured regularly. Echocardiogram was performed. N‐terminal pro‐B‐type natriuretic peptide (NT‐proBNP), high sensitivity troponin T, high sensitivity troponin I, and high sensitivity C‐reactive protein were measured. The major adverse cardiovascular events during hospitalization and follow‐up period were recorded.

**Results:**

Compared with the control group, the heart rate of the test group decreased significantly (*p* < .05). Compared with the control group, the left ventricular end‐diastolic volume and left ventricular end‐systolic volume were significantly decreased while left ventricular ejection fraction was significantly increased in the test group at 90 days after operation. NT‐proBNP of the test group was significantly lower than that of the control group at 7 days after operation (*p* < .05).

**Conclusion:**

For STEMI patients, early use of ivabradine combined with standard therapy such as β‐blocker after successful reperfusion can achieve effective heart rate control, with great safety and tolerance. But the effect of ivabradine on left ventricular remodeling is uncertain.

## 1 INTRODUCTION

Acute myocardial infarction (AMI) is an acute and critical cardiovascular disease. Myocardial infarction causes myocardial scar formation, progressive enlargement and decreased function of left ventricular, leading to ventricular remodeling (Mitchell et al., 1992). Ventricular remodeling is the main cause of heart failure in AMI, affecting the prognosis (Bhatt et al., 2017). Early revascularization is the most effective treatment to prevent ventricular remodeling, and β‐receptor blocker and renin‐angiotensin inhibitor are important drugs for it (Doughty et al., 2004; Pfeffer et al., 1988). For patients with ST‐segment elevation myocardial infarction (STEMI) after emergency percutaneous coronary intervention (PCI), heart rate is closely related to prognosis (Manz et al., (2003)). In the acute phase of myocardial infarction, increased heart rate reduces coronary artery perfusion and myocardial oxygen supply, indicating enhanced sympathetic excitation. Excessive sympathetic activity increases blood pressure and accelerates metabolism, and it induces vascular endothelium to release nitric oxide and other vasoactive substances, resulting in impaired endothelial function and increased permeability as well as increased peripheral vascular resistance. As a result, myocardial ischemia and hypoxia are aggravated, and thus finally promoting ventricular remodeling after myocardial infarction (Antoni et al., 2012). β‐blocker is currently the main drugs to reduce heart rate. However, it has the effects of negative inotropic action, negative conduction, antihypertensive, and it causes many adverse reactions and contraindications. So the application of β‐blocker is greatly limited. Ivabradine is a specific inhibitor of If current in sinus node, which inhibits to reduce sinus node rhythm, thus finally slowing heart rate (DiFrancesco, 2006). Ivabradine, with good safety, has no adverse effects on atrioventricular conduction system, ventricular systolic and diastolic function and blood pressure (Savelieva & Camm, 2008a). Ivabradine is the first drug applied in clinical practice so far to simply reduce the heart rate, especially for patients who are intolerant or are contraindicated to β‐blocker. The SHIFT echocardiography subgroup analysis showed that ivabradine slowed down the heart rate, thus reversing the left ventricular volume in patients with heart failure (Tardif et al., 2011). There are few studies on the application of ivabradine in the acute phase of myocardial infarction, and the benefit of slowing down the heart rate alone is not clear. Therefore, the objective of this study was to investigate whether the early heart rate control using ivabradine combined with β‐blocker on the basis of standardized drug therapy can improve left ventricular remodeling in STEMI patients after successful emergency PCI.

## 2 METHODS

### 2.1 Screening criteria of study subjects

The patients with acute STEMI underwent primary PCI between July 2017 and July 2019 were selected.

The inclusion criteria were as follows: (a) patients with STEMI were diagnosed according to the global definition of AMI (Thygesen et al., (2019)), and electrocardiogram showed continuous elevation of ST‐segment in two adjacent leads two adjacent ST‐segment elevation or new left bundle branch block; (b) patients with successful emergency PCI, and with TIMI flow grade 3 in infarct‐related artery; (c) patients were in sinus rhythm with heart rate ≥ 80 beats/min at 12 hr after PCI.

The exclusion criteria were as follows: (a) patients with unsuccessful PCI, and with TIMI flow grade ≤ 2 in infarct‐related artery; (b) patients with unstable hemodynamics or acute pulmonary edema; (c) patients with atrial fibrillation; (d) patients with the heart rate ≤ 80 beats/min at 12 hr after PCI; (e) patients with symptomatic hypotension; (f) patients with severe liver and kidney function injury. This study was approved by the hospital ethics committee (201,705). Singed informed consent was obtained from each patient.

### 2.2 Research methods

The included patients were randomly divided into the test group and the control group. At 12 hr after PCI, the patients in the test group were given ivabradine (2.5 mg twice a day) combined with metoprolol tartrate (12.5‐25 mg twice a day), while the patients in the control group were given metoprolol tartrate (12.5‐25 mg twice a day) only. The β‐blockers were used in both groups according to the guidelines. Metoprolol tartrate could be replaced with metoprolol succinate (metoprolol sustained‐release tablets) after 2–3 days, and the dose was adjusted in accordance with the condition up to 190 mg/day. In the test group, ivabradine could be increased to 5 mg twice a day according to heart rate up to 7.5 mg twice a day, and at discharge, the resting heart rate was controlled at < 70 beats/min. Before PCI, the patients in both groups were administrated with load dose of 300 mg aspirin and 180 mg ticagrelor/300 mg clopidogrel 300 mg; andⅡb/Ⅲa receptor antagonist (tirofiban), intra‐aortic balloon pump (IABP) and temporary pacemaker implantation were applied according to the condition. PCI was performed according to the guidelines, and thrombus aspiration, balloon dilatation and stent implantation were given to infarct‐related artery as appropriate. The operation was considered successful with TIMI up to grade 3. After operation, all patients were administrated with antiplatelet agents, low molecular weight heparin, angiotensin‐converting enzyme inhibitor (ACEI) or angiotensin receptor blocker (ARB) or angiotensin receptor neprilysin inhibitors (ARNI), statins and other standardized drugs.

### 2.3 Observation indexes

At 1, 7, 30, 90, and 180 days after emergency PCI, all patients were examined with echocardiogram (echo) and N‐terminal pro‐B‐type natriuretic peptide (NT‐proBNP) test. Philips EPIQ 7C was used for echo. The following indexes were measured according to the American Echocardiography Society guidelines (Lang et al., 2015): left ventricular end‐diastolic volume (LVEDV), left ventricular end‐systolic volume (LVESV), and left ventricular ejection fraction (LVEF), left ventricular end‐diastolic diameter (LVEDD), left ventricular posterior wall thickness (LVPWT), interventricular septum thickness (IVST), left ventricular mass (LVM), and left ventricular mass index (LVMI). At 12 hr, and 1, 7, 30 days after emergency PCI, all patients were examined for high sensitivity troponin T (hsTnT), high sensitivity troponin I (hsTnI), creatine kinase‐MB (CK‐MB), and high sensitivity C‐reactive protein (hsCRP). And the heart rate and blood pressure were recorded at admission, before PCI, and 12 hr, 1, 7, 30, 90, and 180 days after PCI. The follow‐up period was 180 days. Follow‐up was performed using outpatient and telephone, so as to observe and record major adverse cardiovascular events, including cardiac death, recurrent myocardial infarction, recurrent target vascular revascularization, re‐admission because of angina pectoris or heart failure. In addition, the adverse drug reactions (ADRs) of ivabradine were also recorded.

### 2.4 Statistical analysis

Statistical analysis was carried out using SPSS20.0 software. The main statistical indexes were tested for normality and homogeneity of variance. The measurement data were expressed as mean ± standard deviation, and *t* test was used for the comparison between groups. Enumeration data were expressed as rates, and chi‐square test was used.

## 3 RESULTS

### 3.1 Screening results of study subjects

**Figure 1:**
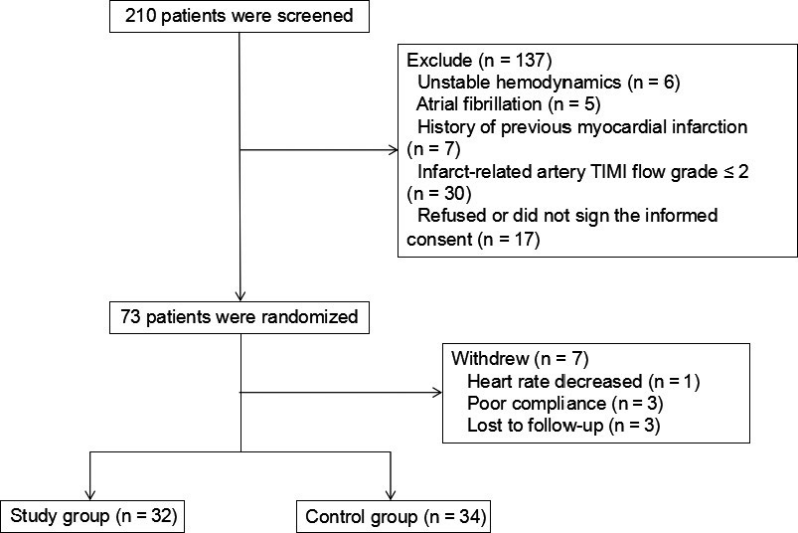
Flow chart of study subject screening

A total of 210 patients were initially selected, and then 137 cases were excluded according to the screening criteria. A total of 73 patients were randomized, and then 7 cases withdrew. Finally, 66 patients were included in this study, including 32 patients in the study group and 34 patients in the control group. The specific screening process is shown in Figure 1.

### 3.2 Baseline characteristics

There was no significant difference in the clinical data between the two groups (all *p* > .05), with comparability (Table 1). In addition, no significant difference was identified in the average oral dose of metoprolol sustained‐release tablets between the two groups. The average oral doses of metoprolol sustained‐release tablets per day in the test group and the control group were 44.53 ± 13.15 mg and 46.10 ± 16.48 mg at discharge, respectively; and 66.80 ± 29.20 mg and 60.77 ± 24.94 mg at 180 days after PCI, respectively (*p* > .05) (Table 1). Meanwhile, the average dose of ivabradine in the test group was 6.13 ± 2.013mg at 180 days after PCI.

**Table 1:** Comparison of the basic characteristics between the two groups

Item	Test group (*n* = 32)	Control group (*n* = 34)	*p*
Age (year)	51.28 ± 9.42	51.50 ± 9.55	.93
Male (*n*, %)	31(96.84)	33(97.06)	.97
BMI (kg/m^2)^	25.33 ± 4.17	25.31 ± 2.91	.98
Smoking (*n*, % )	18(56.25)	22(64.71)	.56
DtoB time (min)	73.38 ± 62.57	66.56 ± 50.38	.63
Hospitalization time (d)	9.59 ± 3.18	11.50 ± 5.02	.07
Complication (n, %)			
Hypertension	13 (40.63)	14 (41.18)	.96
Diabetes	11 (34.38)	8 (23.53)	.33
Hyperlipemia^a^	18 (56.25)	13 (38.24)	.14
Vascular condition (*n*, % )			
Anterior descending branch	23 (71.88)	23 (67.65)	.71
Circumflex artery	3 (9.38)	4 (11.76)	.75
Right coronary	6 (18.75)	7 (20.59)	.85
One‐vessel disease	13 (37.50)	11 (32.35)	.49
Double‐vessel disease	10 (31.25)	14 (41.18)	.40
Three‐vessel disease	9 (28.13)	9 (26.48)	.88
Concomitant medications (*n*, %)			
Aspirin	32 (100)	34 (100.00)	1.0
ADP receptor antagonist	32 (100.00)	34 (100.00)	1.0
ACEI/ARB/ARNI	29 (90.63)	30 (88.24)	.75
Statins	32 (100.00)	34 (100.00)	1.0
Metoprolol succinate dose (mg/d)			
At discharge	44.53 ± 13.15	46.10 ± 16.48	.67
180d after operation	66.80 ± 29.20	60.77 ± 24.94	.37

Abbreviations: ACEI/ARB/ARNI, angiotensin‐converting enzyme inhibitor/angiotensin receptor blocker/angiotensin receptor neprilysin inhibitors; ADP, adenosine diphosphate; BMI, body mass index; DtoB time, door‐to‐balloon time.

^a^ Hyperlipemia: total cholesterol (TC) ≥240 mg/dl (6.22 mmol/L), or low‐density lipoprotein cholesterol (LDL‐C) ≥160 mg/dl (4.14 mmol/L).

### 3.3 Comparison of heart rate and blood pressure between the two groups

**Figure 2:**
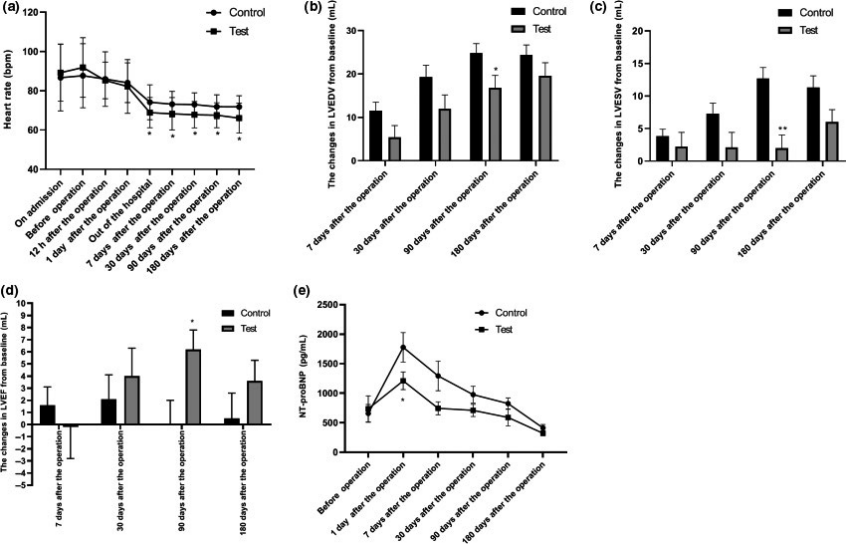
Changes of different indexes between the two groups. A Comparison of heart rate between the two groups. *p < .05 versus control group. B Comparison of changes in LVEDV at various time points from baseline between the two groups. *p < .05 versus control group. LVEDV, left ventricular end‐diastolic volume. C Comparison of changes in LVESV at various time points from baseline between the two groups. **p < .01 versus control group. LVESV, left ventricular end‐systolic volume. D Comparison of changes in LVEF at various time points from baseline between the two groups. *p < .05 versus control group. LVEF, left ventricular ejection fraction. E Comparison of NT‐proBNP levels between the two groups. *p < .05 versus control group

Compared with the heart rate at admission, the heart rate in the both groups was significantly reduced (*p* < .05). Compared with the heart rate in the control group, the heart rate in the test group decreased more significantly at 7 days after operation, at discharge, and at 30, 90, 180 days after operation (*p* < .05) (Figure 2a). No significant difference was identified in blood pressure between the two groups (Table 2).

**Table 2:** Comparison of blood pressure between the two groups

	Systolic blood pressure (mm Hg)			Diastolic blood pressure (mm Hg)		
	Test group	Control group	*p*	Test group	Control group	*p*
On admission	130.97 ± 18.26	130.94 ± 23.58	.996	85.25 ± 13.61	82.82 ± 15.82	.508
12h after operation	113.59 ± 13.09	116.47 ± 16.90	.444	75.06 ± 13.38	78.21 ± 14.63	.367
1 day after operation	108.03 ± 12.72	110.71 ± 15.92	.445	67.34 ± 10.73	71.88 ± 12.70	.123
7 days after operation	109.47 ± 14.91	106.26 ± 15.44	.395	65.28 ± 9.65	66.00 ± 9.88	.766
30 days after operation	110.50 ± 10.23	114.00 ± 17.09	.320	68.63 ± 8.78	71.24 ± 10.64	.283
90 days after operation	108.81 ± 9.88	113.35 ± 12.79	.113	69.56 ± 6.94	71.65 ± 9.23	.306
180 days after operation	110.50 ± 10.04	114.68 ± 10.19	.099	68.41 ± 7.12	71.03 ± 6.56	.124

### 3.4 Comparison of left ventricular remodeling indexes

Echo was used to detect the changes of indexes related to left ventricular remodeling in both groups. As shown in Table 3, compared with the control group, the LVEDV, LVESV, and LVEF of the test group were significantly improved at 90 days after PCI, (*p* < .05). However, there was no significant difference in LVEDV, LVESV, and LVEF between the two groups at 1, 7, 30, and 180 days after PCI (*p* > .05). There was no significant difference in LVMI between the two groups at all time points (*p* > .05) .

**Table 3:** Comparison of echocardiogram between the two groups

	1 day after operation			7 days after operation			30 days after operation			90 days after operation			180 days after operation		
	Test group	Control group	*p*	Test group	Control group	*p*	Test group	Control group	*p*	Test group	Control group	P	Test group	Control group	*p*
LVMI (ml)	94.45 ± 16.95	95.07 ± 18.77	.888	94.65 ± 17.48	96.67 ± 12.70	.605	91.36 ± 16.81	98.13 ± 12.65	.083	93.43 ± 11.84	97.29 ± 9.99	.181	94.14 ± 11.99	95.86 ± 15.39	.632
LVEDV (ml)	95.72 ± 14.20	97.91 ± 16.72	.500	101.10 ± 16.72	108.85 ± 18.02^*^	.085	109.03 ± 17.25^*^	117.62 ± 16.89^*^	.056	112.79 ± 16.28^*^	121.51 ± 13.08^*^	.027	115.23 ± 14.25^*^	122.13 ± 14.99^*^	.069
LVESV (ml)	49.50 ± 10.84	49.03 ± 8.37	.844	51.43 ± 10.80	53.06 ± 9.15	.526	52.73 ± 11.40	56.93 ± 8.28^*^	.108	52.48 ± 11.02	61.13 ± 7.95^*^	.001	55.83 ± 10.79^*^	60.59 ± 9.88^*^	.073
LVEF (%)	48.30 ± 8.78	49.46 ± 8.87	.598	48.35 ± 12.32	51.90 ± 9.00	.354	51.60 ± 8.44	50.86 ± 6.72	.714	53.57 ± 6.40^*^	49.43 ± 6.58	.017	51.47 ± 6.80	49.56 ± 7.98	.315

Note

* P＜0.05 Comparison to baseline within the same group.

Abbreviatios: LVEDV, left ventricular end‐diastolic volume; LVEF, left ventricular ejection fraction; LVESV, left ventricular end‐systolic volume; LVMI, left ventricular mass index.

The values of LVEDV, LVESV, and LVEF were compared with the baseline values in the test group and the control group at 7, 30, 90, and 180 days after PCI (Figure 2b‐d). The increase rates of LVEDV and LVESV in the test group was all lower than that in control group, but only the difference had statistical significance at 90 days after PCI (*p* < .05). In the test group, the LVEF was increased at 30, 90, and 180 days after PCI, but the significant difference between the two groups was only identified at 90 days after PCI (*p* < .05) .

### 3.5 Comparison of laboratory indexes between the two groups

Compared with the control group, the NT‐proBNP level at 7 days after PCI in the test group was significantly reduced (*p* < .05). However, there was no significant difference in NT‐proBNP levels between the two groups before operation, and 1, 30, 90, 180 days after operation (*p* > .05) (Figure 2e). In addition, no significant difference was identified in hsTnT, hsTnI, CK‐MB, and hsCRP between the two group (*p* > .05) (Table 4).

**Table 4:** Comparison of laboratory indexes between the two groups

	Before operation			12h after operation			1 day after operation			7 days after operation			30 days after operation		
	Test group	Control group	*p*	Test group	Control group	*p*	Test group	Control group	*p*	Test group	Control group	*p*	Testgroup	Control group	*p*
TNT (ng/ml)	2.06 ± 2.70	1.35 ± 2.09	.233	5.49 ± 3.47	4.51 ± 3.32	.250	5.79 ± 2.83	5.24 ± 2.92	.449	2.19 ± 2.76	2.54 ± 1.62	.555	0.02 ± 0.03	0.05 ± 0.08	.161
TNI (ng/ml)	19.57 ± 42.23	10.66 ± 14.47	.251	18.09 ± 9.74	22.52 ± 12.33	.387	24.11 ± 14.42	27.44 ± 16.06	.387	5.00 ± 6.27	5.41 ± 6.53	.805	0.09 ± 0.11	0.12 ± 0.16	.448
CK‐MB (U/L)	158.80 ± 185.87	103.44 ± 171.04	.212	247.17 ± 180.63	236.93 ± 224.83	.851	62.29 ± 61.29	92.90 ± 114.36	.212	18.52 ± 13.05	19.13 ± 12.91	.856	14.84 ± 8.64	16.30 ± 6.81	.471
HsCRP (mg/L)	6.06 ± 10.88	5.08 ± 5.19	.713	18.48 ± 22.72	19.70 ± 17.92	.816	30.62 ± 30.19	38.60 ± 33.95	.310	14.01 ± 18.09	19.31 ± 22.31	.310	4.37 ± 9.28	6.26 ± 12.62	.519

Abbreviations: hsTnI, high sensitivity toponin I; hsTnT, high sensitivity toponin T; NT‐proBNP, N‐terminal pro‐B‐ type natriuretic peptide.

### 3.6 Major adverse cardiovascular events and adverse drug reactions in the two groups

During hospitalization and follow‐up period, no cardiac death occurred in both groups. One case in each group was admitted due to recurrent angina pectoris. In addition, 1 patient in the control was readmitted due to heart failure, while 1 patient in the test group had bradycardia (heart rate < 50 bpm). The situation of the latter was improved after ivabradine dose reduction.

## 4 DISCUSSION

Primary PCI is the most effective treatment for STEMI patients, which greatly improves the prognosis. But there is still a high mortality and morbidity. Several studies have suggested that heart rate is an independent predictor of cardiovascular death and all‐cause mortality in STEMI patients after emergency PCI; elevated heart rate increases the risk of death (Kosmidou et al., 2017; Manz et al., 2003; Parodi et al., 2010). The DANAMI‐3 trial (Nepper‐Christensen et al., 2019) has found that early elevated heart rate in STEMI patients is independently associated with myocardial infarct size, decreased ejection fraction, increased all‐cause mortality, and heart failure. The possible mechanisms of increased heart rate affecting the prognosis of AMI are as follows: increases heart rate results in increased myocardial oxygen consumption, shortened diastole, decreased coronary perfusion, and myocardial oxygen supply; meanwhile, the contraction of blood vessels in the acute phase aggravates myocardial ischemia and promotes the expansion of infarction. Increased heart rate also suggests enhanced sympathetic excitation which causes impaired endothelial function and increased permeability as well as increased peripheral vascular resistance. As a result, myocardial ischemia and hypoxia are aggravated, which promotes the apoptosis of myocardial cells and induces myocardial fibrosis, and thus finally causing left ventricular remodeling (Ambrosetti et al., 2017; Inoue et al., 2013). Therefore, we believed that the lower heart rate after myocardial infarction is conducive to the angiogenesis and the establishment of collateral circulation in the infarct and its surrounding area (Mulder & BarbierS, 2004; SchirmerSH, et al., 2009 ^).^ The decrease of heart rate can increase diastole and facilitate the filling of the ventricles, thus improving the perfusion of the coronary arteries, and reducing the load of the heart. Therefore, cardiac function is restored and the prognosis is improved.

β‐blocker has a positive effect on reducing the mortality of acute STEMI, so the STEMI patients without contraindications should take oral β‐blocker within 24 hr after onset (Ibanez & Agewall, 2017). However, β‐blocker has the effects of negative inotropic action, negative conduction, and antihypertensive, and it causes many adverse reactions and contraindications, so its application is limited in clinical practice. In clinical practice, it is significantly underused and underdose in STEMI patients, causing higher difficulty in early heart rate control.

Ivabradine is a specific inhibitor of the If current in the sinus node. It inhibits the If pacing current to delay the rate of diastolic depolarization in the sinus node and to reduce sinus node rhythm, so as to finally slow heart rate (Thygesen et al., 2019). Ivabradine, as a new drug to lower sinus rate, has no negative inotropic action, no negative conduction effect, and no effect on blood pressure (Savelieva & Camm, 2008b). The SHIFT echocardiography subgroup analysis (Tardif et al., 2011) showed that ivabradine slowed down the heart rate while reversing the left ventricular remodeling in heart failure patients with reduced ejection fraction, thereby improving heart function and long‐term prognosis.

Animal experiments on AMI (Heusch & skyschally A et al., 2008; Mulder & BarbierS, 2004; O'Connor et al., 2016) showed that ivabradine slowed heart rate to improve the local blood flow in ischemic myocardium and systolic function, and thus reducing myocardial ischemic area. Priti et al. (Priti et al., 2017) found that ivabradine is as effective as metoprolol in inferior AMIT patients, reducing the risk of first‐ or second‐degree atrioventricular block. Fasullo et al. (FasulloS & MaringhiniG, 2009) revealed that after successful PCI, ivabradine was given to the anterior STEMI patients with impaired left ventricular function and rapid heart rate; ivabradine significantly improved LVEF compared with metoprolol after 2‐month follow‐up. Edouard et al. (EdouardG et al., 2014) showed that STEMI patients with successful reperfusion were treated with ivabradine on the basis of standard treatment, and cardiac MRI showed the significant improvement in myocardial remodeling.

This study is a single‐center prospective randomized controlled trial. The same dosage of β‐blocker was applied in the test group and the control group. In the test group (ivabradine combined with β‐blocker), the heart rate was significantly reduced in STEMI patients after emergency PCI, and this advantage occurred in the early stage and lasted until the end of the study. This result indicates that ivabradine could bring benefits independent of β‐blocker and did not affect the use of β‐blocker. There was no difference in blood pressure between the two groups, indicating that ivabradine did not affect blood pressure. Francesco et al. (Francesco & GiuseppeP, 2016) proved that ivabradine was also safe and effective for patients with STEMI and cardiac shock.

Compared with the control group, the LVEDV, LVESV, and LVEF in the test group were significant improved at 90 days after PCI, and NT‐proBNP was significantly reduced at 7 days after PCI (*p* < .05). These results suggested that ivabradine combined with β‐blocker performed early control of heart rate to improve left ventricular remodeling in the patients with myocardial infarction. However, at 180 days after operation, although LVEDV and LVESV in the test group tended to be improved compared with the control group, there was still no significant difference (*p* > .05); LVEF also failed to show improvement. These results indicated that the benefit of ivabradine at 90 days after operation on the remodeling was not sustained. So there is limited evidence that ivabradine improve left ventricular remodeling through early heart rate control in STEMI patients after emergency PCI, and the efficacy of ivabradine is uncertain. Because the follow‐up was not long enough, we continued this study. But the false‐negative results may be presented due to the small sample size. During the 180‐day follow‐up, there was no difference in major cardiovascular events between the two groups. Only one patient in the test group developed bradycardia, and the condition was improved after dose reduction. The result of follow‐up suggests that ivabradine is safe and well tolerated. In addition, LVEDV and LVESV were progressively expanded in STEMI patients and remodeling after myocardial infarction still occurred even after successful reperfusion and standardized drug treatment, which started as early as 7 days after operation.

This study is a nondouble‐blind study, with small sample size and limited follow‐up time, which is only a pilot study. If the mean and variance of the LVEF of the test group and the control group at 180 days after operation are selected and the sample size is estimated using SPSS20.0 software, the total sample size should be 1,088 cases to obtain the statistically significant results of the two groups. Therefore, a larger sample size test is required to verify whether the early use of ivabradine can improve left ventricular remodeling and can protect heart function after successful reperfusion in STEMI patients, and to verify whether ivabradine can improve clinical outcomes. In addition, some issues have yet to be clarified, such as whether the clinical benefit of ivabradine is different between acute anterior and inferior myocardial infarction, or whether the efficacy of ivabradine, such as TIMI flow ≤ 2 is equal in the patients with unsuccessful reperfusion? In this test, the resting heart rate at discharge was controlled at < 70 bpm. Is it more beneficial if the heart rate control is more stringent, such as around 60 bpm? All of these questions are required further investigation and longer follow‐up.

## 5 CONCLUSION

Ivabradine simply reduces heart rate but not affecting myocardial contractility, blood pressure, and cardiac conduction function, leading to the easy, safe, and tolerable way for early heart rate control in AMI. After emergency PCI in STEMI patients, the effect of early combined use of ivabradine (12 hr after operation) on the basis of conventional drug therapy (including β‐blocker) on left ventricular remodeling is uncertain, and there may be some improvement. Further multi‐center and large‐sample studies are expected.

## ETHICAL APPROVAL

This study was approved by the hospital ethics committee (201,705).

## CONFLICT OF INTERESTS

The authors claim that there is no conflict of interest between them.

## Data Availability

Data available in Article.
